# Incidence and Clinical Impacts of COVID-19 Infection in Patients with Hemodialysis: Systematic Review and Meta-Analysis of 396,062 Hemodialysis Patients

**DOI:** 10.3390/healthcare9010047

**Published:** 2021-01-05

**Authors:** Chun-Yu Chen, Shih-Chieh Shao, Yih-Ting Chen, Cheng-Kai Hsu, Heng-Jung Hsu, Chin-Chan Lee, Chiao-Yin Sun, Yung-Chan Chen, Ming-Jui Hung, I-Wen Wu

**Affiliations:** 1 Department of Nephrology, Chang Gung Memorial Hospital, Keelung 204, Taiwan; shone@cgmh.org.tw (C.-Y.C.); b9402031@cgmh.org.tw (Y.-T.C.); kylegb@cgmh.org.tw (C.-K.H.); r5267@cgmh.org.tw (H.-J.H.); leefang@cgmh.org.tw (C.-C.L.); sun3970@cgmh.org.tw (C.-Y.S.); 2 College of Medicine, Chang Gung University, Taoyuan 333, Taiwan; cyc2356@cgmh.org.tw; 3 School of Pharmacy, Institute of Clinical Pharmacy and Pharmaceutical Sciences, College of Medicine, National Cheng Kung University, Tainan 701, Taiwan; scshao@cgmh.org.tw; 4 Department of Pharmacy, Keelung Chang Gung Memorial Hospital, Keelung 204, Taiwan; 5 Department of Nephrology and Kidney Research Center, Chang Gung Memorial Hospital, Linkou 333, Taiwan; 6 Department of Cardiology, Chang Gung Memorial Hospital, Keelung 204, Taiwan; hmj1447@cgmh.org.tw

**Keywords:** COVID-19, hemodialysis, incidence, meta-analysis, mortality, systematic review

## Abstract

Hemodialysis (HD) patients are highly susceptible to COVID-19 infection. However, comprehensive assessments of current evidence regarding COVID-19 in HD patients remain incomplete. We systematically searched PUBMED and EMBASE for articles published on incidence or mortality of COVID-19 infection in HD patients until September 2020. Two independent researchers extracted data and study-level risk of bias across studies. We conducted meta-analysis of proportions for incidence and mortality rate. Study heterogeneity and publication bias were assessed. A total of 29 articles with 3261 confirmed COVID-19 cases from a pool of 396,062 HD patients were identified. Incidence of COVID-19 in these HD patients was 7.7% (95% CI: 5.0–10.9%; study heterogeneity: I2 = 99.7%, *p* < 0.001; risk of publication bias, Egger’s test, *p* < 0.001). Overall mortality rate was 22.4% (95% CI: 17.9–27.1%; study heterogeneity: I2 = 87.1%, *p* < 0.001; risk of publication bias, Egger’s test: *p* = 0.197) in HD patients with COVID-19. Reported estimates were higher in non-Asian than Asian countries. Quality of study may affect the reported incidence but not the mortality among studies. Both incidence and mortality of COVID-19 infection were higher in HD patients. Available data may underestimate the real incidence of infection. International collaboration and standardized reporting of epidemiological data should be needed for further studies.

## 1. Introduction

The novel coronavirus, COVID-19, continues to generate a tremendous global burden with 62,363,527 confirmed cases and 1,456,687 deaths worldwide, as of the end of November 2020 [1]. Many risk factors related to the incidence of COVID-19 infection have been identified; for example, advanced age, diabetes mellitus, hypertension and smoking [2,3]. In addition, the mortality rate of COVID-19 infection varies from country to country, ranging from 35.4 per million population (South-east Asia) to 904.4 per million population (America) [1]. Higher risks of mortality caused by COVID-19 are found in patients with older age, more comorbidities, and immune dysfunction [3,4]. However, a comprehensive assessment of published literature with regard to the epidemiology in patients with renal impairment infected by COVID-19 remains scarce. 

The prevalence of chronic kidney disease (CKD) is about 9.1% worldwide [5], and end-stage renal disease (ESRD) is associated with higher comorbidity, mortality risks, and socioeconomic impacts, affecting 2,859,750 patients globally [6]. In-center hemodialysis (HD) is the predominant renal replacement modality across different countries, except in Hong Kong, Mexico, and Guatemala [7]. HD patients are immune-dysregulated patients on account of uremia, comorbidities, and dialysis procedure-related bio-compatibility [8]. Furthermore, frequent personnel contact in crowded areas for their in-center facility treatment may hamper effective protective measures (such as social distancing, reducing personal contact, staying home) against COVID-19 infection. CKD has previously been associated with increased risk for COVID-19 infection [9], but the published observational studies have identified varying incidence and clinical impacts of COVID-19 in HD patients. To the best of our knowledge, there is no comprehensive understanding of incidence and clinical outcomes of COVID-19 in HD patients, but this information could be beneficial for the development of proper screening or preventive strategies against COVID-19 infection in this vulnerable population. The aim of the present study is to fill this knowledge gap, and systematically quantify the incidence and clinical impacts of COVID-19 infection in HD patients, identity methodological bias, and estimate the influence of bias on the results of studies. 

## 2. Materials and Methods 

### 2.1. Design and Search Strategy 

We conducted a systematic review and meta-analysis of observational studies in published literature from PUBMED and EMBASE until 9 September 2020. The MeSH or Emtree terms included COVID-19, hemodialysis, incidence, prevalence, mortality, or prognosis (Supplementary Table S1). Studies were required to provide data on either incidence of proven COVID-19 infection (such as nucleic acid testing by polymerase chain reaction, serology, or image study) or related mortality of patients receiving maintenance HD therapy. The reference lists of included articles were also hand-searched. References were managed using EndNote version X8, Clarivate Analytics, PA, USA. This study adhered to the reporting guidelines of Preferred Reporting Items for Systematic Reviews and Meta-analyses (PRISMA) [10] (Supplementary Table S2), and it has been recorded in the International Prospective Register of Systematic Reviews (PROSPERO) database (CRD42020209134).

### 2.2. Literature Selection

Two independent researchers (CYC and IWW) screened titles and abstracts to identify potentially eligible studies for full-text review. Original articles and case series with over 2 cases in English language were reviewed. We excluded the articles reporting incomplete data on incidence or mortality.

### 2.3. Data Extraction and Study Quality

Data extraction was completed in duplicate by 2 independent reviewers (CYC, WIW). When multiple articles reporting data from the same study population were identified, the most comprehensive data were used. Information including country, study design, settings, age, gender, presenting symptoms or signs, laboratory findings, treatment, and preventive strategies were extracted. The raw numbers of total, infected, and deceased cases were accurately recorded. Incidence rates were estimated by calculating the affected cases from the overall HD patients infected by COVID-19. Mortality was expressed as case fatality rate. We contacted the study authors regarding possible incomplete data on incidence or mortality presented in selected publications.

The methodological quality of included studies was assessed independently by 2 authors (YTC, CKH) based on a 20-item critical appraisal checklist for case-series studies developed by the Institute of Health Economics (IHE) [11]. If we answered ‘yes’ on an item of the checklist, then the item scored 1 point. If we answered ‘no’ or ‘unclear’ on a checklist item, then 0 points were scored. We considered studies scoring 14 or more points (≥70%) as “good quality” [12]. When the reviewers’ assessments differed with regard to data extractions or study quality evaluations, the additional reviewer (SCS) were drawn in on a case by case basis to discuss and make the final judgments. 

### 2.4. Data Synthesis and Statistical Analysis

We performed meta-analysis of the proportions (with 95% confidential interval, CI) for the incidence and mortality rates of COVID-19 infection in patients receiving HD. Statistical heterogeneity among the studies was measured by Cochran’s Q test with the p-value, and the extent of heterogeneity attributable to heterogeneity was measured by the I^2^ statistic. We planned subgroup analyses based on geographic area (e.g., Asia or non-Asia) and quality of study (e.g., good or poor). We also used Egger’s test to determine potential publication bias. Statistical analyses were performed using MedCalc for Windows, version 15.0 (MedCalc Software, Ostend, Belgium).

## 3. Results

**Figure 1:**
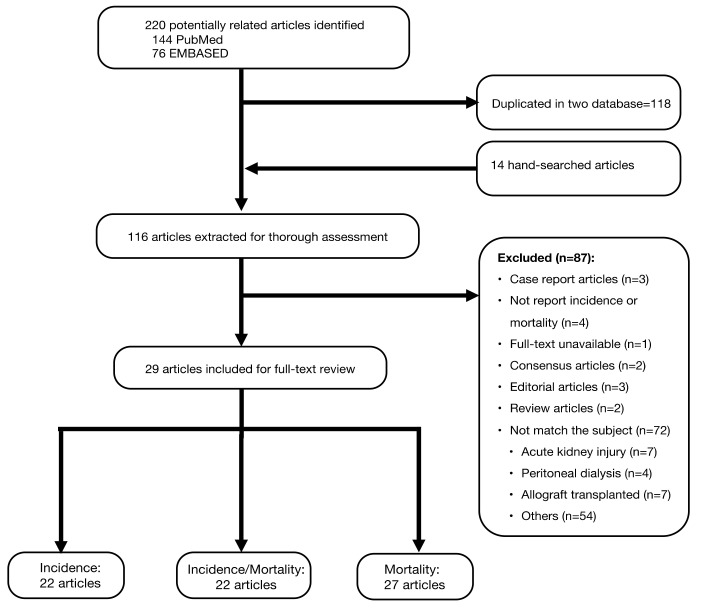
Flow chart of literature search and selection.

We identified 220 records form PUBMED (*n* = 144) and EMBASE (*n* = 76) databases for the initial assessment, and only 29 articles were included for full-text review (Figure 1). This systematic review and meta-analysis evaluated 29 international studies with 3261 confirmed COVID-19 cases from a pool of 396,062 HD patients. The mean age of COVID-19 infected HD patients was 64.9 years and 64.5% were men. The characteristics of the included studies are described in Table 1. Due to emergency and the uncertainty of this novel disease, articles were emerging into the literature after short observation periods. The mean observation time was 46.6 days (ranging from 13 to 121). We identified 22 HD cohorts (Asian countries: 47.8%; good study quality: 43.5%) for assessment of incidence and 27 HD cohorts (Asian countries: 42.9%; good study quality: 35.7%) for analysis of mortality related to COVID-19 infection (Table 2). 

**Figure 2:**
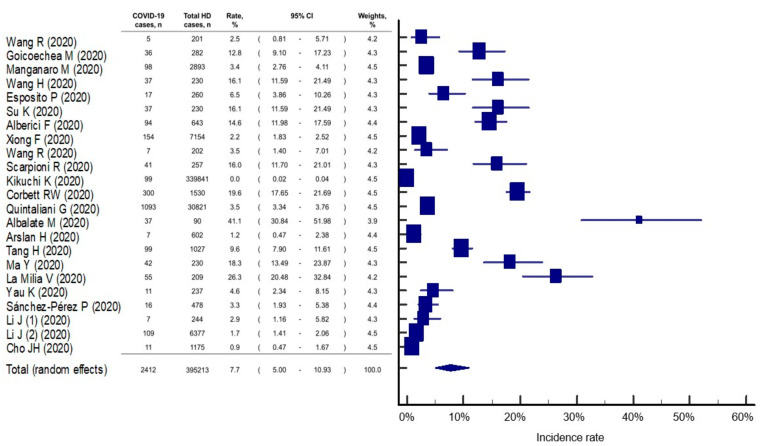
Incidence rate of COVID-19 infection in patients with hemodialysis. The study of Li J et al [13]. reported two independent cohorts that were analyzed separately for meta-analysis. HD, hemodialysis; CI, confidential interval.

We found that the incidence of COVID-19 infection in patients receiving HD therapy was 7.7% (95% CI: 5.0–10.9%), but there was evidence of statistical heterogeneity among the studies (I^2^ = 99.7%, *p* < 0.001) (Figure 2). Egger’s test (*p* < 0.001) indicated a high risk of publication bias. Our meta-analysis also showed that the incidence of COVID-19 in HD patients was 5.0% (95% CI: 2.5–8.4%) and 10.5% (95% CI: 6.6–15.3%) in Asian and in non-Asian countries, respectively. In studies with good quality, the incidence was estimated at 5.2% (95% CI: 1.2–11.8%) of HD patients infected by COVID-19, but it was lower than in those studies with poor quality (8.7%, 95% CI: 6.4–11.2%). 

Fever was the most predominant clinical manifestation (reported in 19 studies) and was observed in 889 of 1448 COVID-19 infected HD patients (61.4%, 95% CI: 40.2–65.5%), followed by cough (19 studies, 654 of 1398 patients, 46.8%, 95% CI: 25.7–44.7%), dyspnea (16 studies, 438 of 1246 patients, 35.2%, 95% CI: 16.9–36.6%) and fatigue (12 studies, 136 of 471 patients, 35.2%, 95% CI: 14.6–49.9%). Eleven studies reported hematological parameters of infected patients; however, most of them had white blood cells, lymphocytes, neutrophils and platelets within normal ranges (Table 1). From 16 studies reporting the presence of dyspnea or 11 studies reporting the use of chest radiography or tomography, only 12 studies have provided image descriptions. Bilateral lungs involvements with ground-glass opacities were the most common finding (11 studies, ranged from 43 to 100% of study population). However, negative radiological findings can also be found in 10–50% of HD patients [33,41].

**Figure 3:**
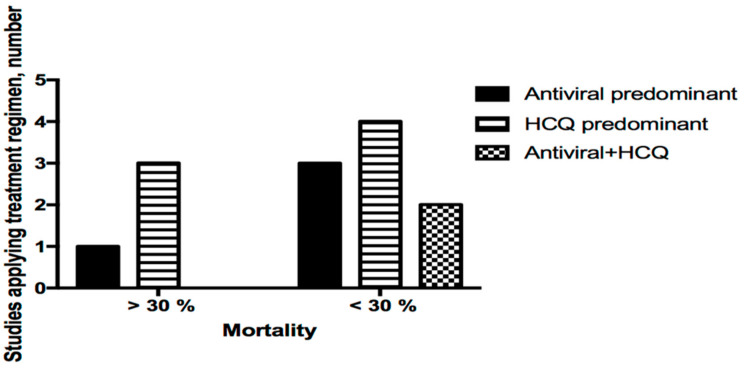
Treatment regimen used and proportion of high mortality (>30%) in different studies. Three main patterns were identified among 11 studies. Chi-square test, *p* = 0.587.

The treatment regimen for COVID-19 infection in HD patients is largely empirical and has been incomplete in most of the studies. Comparison of therapeutic effects between different treatment regimens was extremely difficult because information regarding the dosing, duration, and indication of prescription has been largely unknown among studies. Eleven studies reported the use of antiviral agents and hydroxychloroquine, seven studies reported the use of tocilizumab, and nine studies reported the use of corticosteroid. From the reported data, we identified three main treatment regimens: antiviral agent predominant, hydroxychloroquine predominant, or combination of antiviral agent plus hydroxychloroquine. Although reduced use of these regimens was more likely to have higher mortality (mortality > 30%, Figure 3), the exact effect remains to be proven in well-designed clinical trials. 

Inpatient care was needed in 1045 (82.5%) of 1267 patients, from 19 studies pooled. Moreover, 11 of these 19 studies required absolute hospitalization (100.0%) of their infected patients. Admission to intensive care unit occurred in 84 (6.6%) of patients. The mean in-hospital length of stay was 14.5 ± 8.8 days (data available for analysis from 8 studies). Sixteen studies described presence of the acute respiratory distress syndrome (ARDS). The syndrome was found in 133 of 717 infected cases (18.5%, 95% CI 4.5–21.7%). However, only six studies reported the number of patients using mechanical ventilator supports. The proportion of patients needing support ranged from 0 to 93% according to different studies.

**Figure 4:**
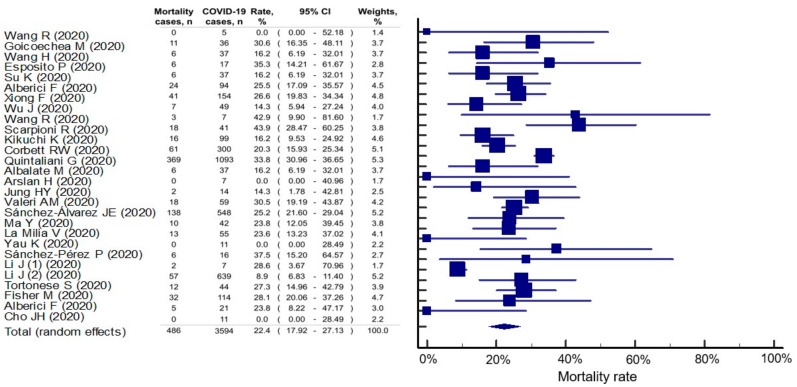
Mortality rate in hemodialysis patients with COVID-19 infection. The study of Li J et al [13]. reported two independent cohorts that were analyzed separately for meta-analysis. The second cohort of Li J et al. reported mortality in 57 deaths from 639 COVID-19 cases (all cases showed feature of viral pneumonitis and 109 were further confirmed by nuclear acid testing). HD, hemodialysis; CI, confidential interval. HD, hemodialysis; CI, confidential interval.

The overall mortality rate in HD patients with COVID-19 was 22.4% (95% CI: 17.9–27.1%), but significant statistical heterogeneity among the studies was found (I^2^ = 87.1%, *p* < 0.001) (Figure 4). However, based on the results of Egger’s test (*P* = 0.197), there was no publication bias in this outcome. Compared with those in Asian countries (17.0%, 95% CI: 11.4–23.5%), COVID-19-infected HD patients in non-Asian countries had a higher mortality rate (26.7%, 95% CI: 22.5–31.0%). In the studies with good quality, mortality was estimated at 23.8% (95% CI: 20.2–27.6%), which was similar in those studies with poor quality (21.6%, 95% CI: 14.5–29.6%). 

The causes of mortality were unreported in most of the studies. Only seven studies reported cause of mortality. The respiratory failure secondary to ARDS (63–100%) was the main cause of mortality, followed by cardia arrest, sepsis, and hyperkalemia.

**Figure 5:**
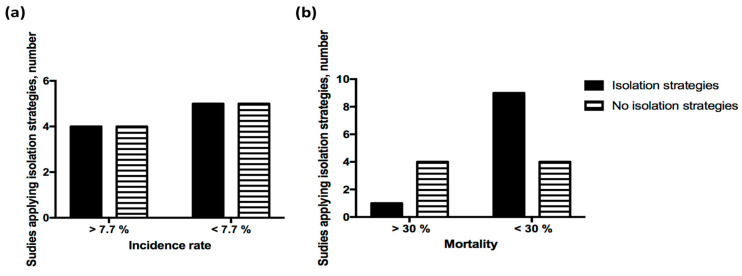
Impact of use of isolation strategies on the outcome of COVID-19 infection in HD patients. The impacts of applying isolation strategies at HD facilities on the (**a**) incidence (*p* = 1.0) and (**b**) mortality (*p* = 0.088) of COVID-19 was not significant. *p* value between groups by using Fisher’s exact test.

Twenty studies described the preventive strategies implemented for their HD patients. Ample alerts were observed in these studies regarding the use of protective measures. Masking was mandatory in the vast majority of HD facilities. Other preventive methods included the use of gloves, face shields, disposable gowns, caps, or alcohol sanitizer (Table 2). Isolation in independent areas of treatment was instructed rather than social distancing in some studies. However, the implementation of isolation of patients in dedicated areas had small influence in the incidence (Figure 5a) or mortality (Figure 5b) of HD patients.

## 4. Discussion

The COVID-19 infection has been declared a global emergency affecting 0.7% of the 7.8 billion worldwide human population, with the burden still growing [1]. In spite of universal precautions adopted to prevent this infection in the HD community, the incidence of this novel viral infection remains high among HD patients. This systematic review and meta-analysis of 29 international studies, including 3261 confirmed COVID-19 cases, drawn from a pool of 396,062 HD patients, found that the incidence of COVID-19 infection was 7.7% and the mortality rate was 22.4%, i.e., higher than in the general population. Understanding of incidence, clinical presentation, and mortality related to COVID-19 in HD patients may help to design appropriate interventions for prevention, timely diagnosis, and treatment of this global challenge in this vulnerable population.

HD patients are more susceptible to COVID-19 infection because of greater age, coexistence of comorbidities, and immune-suppressed status [15,16]. Necessary, frequent visits to areas of high population density (public transportation or HD facilities) and close personal contacts (with medical, nursing, or caregiver staff) make effective strategies to prevent viral infection, such as social distancing or stay-home orders, difficult to implement for this select population of patients [28]. Accordingly, a 15.4-fold increase was noted in the incidence of COVID-19 in our study, with patients also being older compared to the general population [39]. The mean ages of patients was slightly greater (63.5 years) in patients of non-Asian studies than those of Asian studies (61.8 years). Variations in both criteria for viral screening and confirmatory methods of COVID-19 infection may also explain the difference in the incidence observed between studies of the two geographic areas. The difference in incidence observed between Asian and non-Asian populations may be greater than expected. Asian countries adopted universal screening using a nucleic acid test, serology, or computed tomography. The serologic antibody response is detectable 7 to 10 days or later after the onset of symptoms of COVID-infection in the general population [42]; however, the humoral response may extend from 14 to 55 days in HD patients [21]. By contrast, non-Asian countries, except for Canada [14], conducted viral screening only in symptomatic patients or patients at high risk of exposure, using mainly the nucleic acid test. The latter approach may mitigate the overwhelming burden on testing facilities; however, subclinical cases increase the difficulty of identifying COVID-19-infected HD patients and controlling outbreaks in the dialysis centers, which may lead to underestimation of the exact incidence of COVID-19 infection in asymptomatic HD patients. Manganos et al., at a very early stage of the disease outbreak, used radiographic signs suggestive of interstitial pneumonia as surrogate criteria for COVID-19 disease [25]. A nationwide serology screening involving 28,503 HD patients in the US found that seroprevalence was 8.3%, standardizing with the US dialysis population [43]; however, serology data were largely unreported in non-Asian studies. All these differences may confer heterogeneity to the global incidence observed in the HD population. 

COVID-19-related mortality estimates range from 1.4% to 8% in the general population and are higher (25.5–39%) in hospitalized patients [2,3,36,44,45]. The prognosis of HD patients with COVID-19 remains unclear. We found overall mortality of 22.4% in HD patients infected with COVID-19. Previous literature has indicated several risk factors for high mortality in HD, including greater age, male gender, underlying cardiac or pulmonary disease, diabetes and hypertension and the use of mechanical ventilation [15,16,25]. Cough was associated with risk of mortality in French and Italian HD patients [23,34], fever predicted mortality in an Italian HD cohort [28]. Other prognostic factors have included dialysis vintage, thrombocytopenia, lymphopenia, and increased LDH or CRP level [23,28,36]. However, most studies have reported less severe clinical symptoms in HD patients compared with the general population [13,19,20,36]. In a Chinese series, the most common symptoms were fever, cough, and bilateral ground-glass or patchy opacity of the lungs [21]. However, a retrospective Chinese study comparing 49 HD vs. 52 non-renal failure patients having similar baseline characteristics found that fever, fatigue, and dry cough were more predominant in controls but less frequent in HD infected patients. In this series, fatigue and anorexia were the most common symptoms among HD-infected patients [18]. In addition, 25% of infected patients confirmed by nucleic acid test and 79% of those identified by serologic testing were asymptomatic during the whole clinical course [21]. Further large prospective studies, including different ethnicities, should be conducted to inform risk stratification with the ultimate goal of improving the outcome of HD patients with COVID-19 infection.

This viral infection can trigger severe immune cytokine storm, ARDS, and respiratory failure, leading to high risk of mortality [46]. Increased serum concentration of interleukin (IL)-2, IL-6, IL-7, granulocyte-colony stimulating factor, interferon-γ inducible protein 10, monocyte chemoattractant protein-1, macrophage inflammatory protein 1-α, tumor necrosis factor (TNF)-α, and ferritin have been observed in individuals infected with COVID-19 [46,47]. This hyper-inflammatory storm may play a role in the tissue damage and death of patients [4,48]; however, this response is blunted in infected HD patients. Several studies have revealed leukopenia, lymphopenia, lower serum calcium concentration, and elevated CRP levels in HD patients; however, other researchers have failed to find changes in numbers of granulocytes or lymphocytes in infected HD patients [17,18,19,21,22]. Ma Y et al. found that the counts of T cells, CD4 T cells, CD8 T cells, natural killer cells, and B lymphocytes were reduced in the peripheral blood of infected HD patients compared with non-HD patients. In contrast, the serum levels of IL-4, IL-6, IL-10, interferon-γ, and TNF-α were lowest in infected HD patients compared to non-infected HD patients or COVID-19-infected patients with normal renal function [20]. Further evidence of attenuated cytokine reaction in HD patients could be manifest in the low proportion of ARDS reported in various studies. Our meta-analysis indicated an overall incidence of ARDS of 18.5%, which is significantly lower than the reported incidence from hospitalized patients (33%) [49]. On the other hand, the reduction of activity of angiotensin-converting enzyme 2 (ACE2) was observed in HD patients compared to CKD or renal transplant recipients [50]. This transmembrane protein is the key factor for the COVID-19 virus entering the host cell [51]. Theoretically, the binding of viral particle to ACE2 induces the downregulation of this enzyme, increasing levels of bradykinin and its metabolite, the des-Arg(9)-bradykinin. In turn, this dysregulated bradykinin signaling upregulates pro-inflammatory genes and nuclear death signaling [52]. It is unknown whether the immuno-compromised status per se, the dysregulated bradykinin axis, or the hemodiafiltration/hemoperfusion may have facilitated cytokine clearance. Although these findings may prove beneficial for patient survival, they also imply protracted duration in eliminating the virus and hence persistent shedding in HD patients, which must be considered from a public health perspective. Studies investigating the dynamics of viral load in HD patients remain limited. Appropriate duration for quarantine or treatment course should be designed in future trials to avoid an inadvertent transmission of COVID-19 among HD patients.

Again, studies among HD patients from Asian countries have reported lower mortality (17.0%) than from non-Asian countries (26.7%). Asian patients are more likely to be young and have milder symptomatology than their non-Asian counterparts. The ubiquitous deployment of CT scan, especially in China, may have allowed better detection of severe lung condition feasible to timely intervention [22]. The optimal antiviral therapy for HD patients is largely unknown. Current consensus recommends the use of antiviral therapy in the first stage for viral clearance, followed up by immune-suppressive strategies (glucocorticoids or anti-cytokine drugs) to ameliorate cytokine injury [24]. Combinations of antibiotics or Chinese herbal medicine administrations were observed in Chinese studies [17,18,19]. We could not draw any solid conclusion from comparison of treatment regimen to patient outcome in our study. Further randomized controlled trials comparing the effectiveness and safety of different therapies should be undertaken in HD patients. 

The findings of our study have several implications for clinical practice and also preventive medicine. The high incidence and subclinical presentation may prevent the timely identification of infected patients and may result in extensive spreading of virus in highly loaded medical areas. Universal testing to stop the dissemination of COVID-19 should be leveraged with the appropriate testing capacity. For infected HD patients, cautions regarding prolonged viral shedding and prudence in the use of immuno-suppressive agents should be considered, taking into account the blunted immune reaction of HD patients. Ultimately, given the multiple coexistent high-risk conditions, vaccination, if proven safe, should be prioritized for HD patients.

The results of our study provide a panoramic understanding of COVID-19 infection in HD patients. However, several limitations should be addressed. First, COVID-19 infection is unlikely to be eliminated in the near future, and more studies related to the epidemiology in HD patients with COVID-19 infection will be published after the presented work. Therefore, a regularly updated systematic review and meta-analysis is suggested to confirm our findings. Second, all included studies report mortality with COVID-19 infection in HD patients after short follow-up periods, while the long-term outcomes in this population are yet to be determined. Third, we could not derive all the important information from the included studies, even if we did contact the study authors for those data. To reduce the effect of possible reporting bias on our result estimates, we conducted subgroup analyses using the study quality, which showed similar findings to the overall analyses. Finally, we included studies reporting data of patients receiving in-center HD treatment. Data of dialysis patients undergoing different modalities, such as home-HD or peritoneal dialysis, remain unknown. We suggest the introduction of standardized international registry of COVID-19 infected dialysis patients to collect detailed patient characteristics and prognosis data, which would be beneficial for the fight against the current pandemic and for the further development of optimal management for dialysis patients. 

## 5. Conclusions

This systematic review and meta-analysis of international studies has demonstrated a higher incidence of COVID-19 infection and related mortality among HD patients compared to the general population. Available data may underestimate the real incidence of infection because of subclinical presentation and screening criteria used for diagnosis. In spite of differences in incidence and mortality observed between Asian and non-Asian infected HD patients, the present data may provide insight for the design of surveillance and diagnosis strategies specific to HD patients. International collaboration and standardized reporting should be urgently initiated to refine consensus on the optimal management of this novel infection in dialysis patients.

## Figures and Tables

**Table 1:** Characteristics of studies reporting COVID-19 infection in hemodialysis patients.

First Author	Country	Total HD, *n*	COVID-19, *n*	Age (Covid-19)	Male, % (Covid-19)	Presenting Symptoms/Signs, *n* (%)						Laboratory Findings, Median (IQR) or Mean ( ± SD ), 10⁹/L				Treatment, *n* (%)				Location of Treatment, *n* (%)			ARDS
						Fever	Fatigue	Cough	Dyspnea	GI	Myalgia	WBC	Lymphocytes	Neutrophils	Platelet	Antiviral	HCQ	Tocilizumab	CS	In	Out	ICU	*n*, %
Yau K	Canada	237	11	66 (63–72)	6 (55)	1 (9)	N/A	3 (27)	0 (0)	N/A	N/A	4.72 (3.1–21.8)	0.54 (0.05–1.38)	N/A	N/A	N/A	N/A	N/A	N/A	5 (45)	6 (55)	2 (18)	0
Wang H	China	230	37	N/A	N/A	N/A	N/A	N/A	N/A	N/A	N/A	N/A	N/A	N/A	N/A	N/A	N/A	N/A	N/A	37 (100)	0	0	0
Su K	China	230	37	N/A	N/A	N/A	N/A	N/A	N/A	N/A	N/A	N/A	N/A	N/A	N/A	N/A	N/A	N/A	N/A	N/A	N/A	N/A	0
Xiong F	China	7154	154	63.2 (13.1)	75 (57.3)	68 (52) a	59 (45) a	49 (37) a	34 (26) a	35 (27) a	NA	5.0 (3.8–7.3)	0.7 (0.5–1.1)	3.9 (3.0–6.1)	144.2 (107–186)	92 (80) c	N/A	N/A	19 (17.3) d	N/A	N/A	N/A	16 (10)
Wu J *	China	49	49	62 (54–71)	31 (63)	23 (47)	29 (59)	24 (49)	22 (45)	6 (12)	NA	5.6 (4.7–7.6)	0.8 (0.5–1.0)	4.0 (3.1–5.6)	169 (120–234)	47 (96)	N/A	N/A	8 (16)	N/A	N/A	N/A	10 (20)
Li J (1)	China	244	7	59 (39–66)	4 (57)	1 (14)	0 (0)	0 (0)	0	0	0	5.4 (2.6–6.4)	0.5 (0.4–0.9)	4.4 (1.9–5.0)	134.0 (23.3)	0	0	0	0	7 (100)	0 (0)	N/A	N/A
Li J (2)	China	6377	109	N/A	N/A	N/A	N/A	N/A	N/A	N/A	N/A	N/A	N/A	N/A	N/A	N/A	N/A	N/A	N/A	N/A	N/A	N/A	N/A
Wang R	China	201	5	57.6 (47–67)	3 (60)	3 (60)	3 (60)	1 (20)	2 (40)	2 (40)	0	7.50 (5.94–9.25)	0.80 (0.56–0.88)	5.69 (4.97–7.76)	NA	N/A	N/A	N/A	N/A	5 (100)	0	0	0
Ma Y	China	230	42	64.57 (47–76)	25 (60)	4 (10)	3 (7)	3 (7)	N/A	2 (5)	N/A	N/A	1.42 (0.85–1.56)	4.92 (4.23–7.06)	154 (140–200)	N/A	N/A	N/A	N/A	32 (76)	10 (24)	3 (7)	2 (5)
Tang H ^	China	1027	99	61.3 ± 13.8	55 (56)	27 (27)	N/A	27 (27)	14 (14)	11 (11)	N/A	4.9 (4.04–6.51)	0.86 (0.66–1.15)	3.45 (2.87–4.52)	161 (117–200)	N/A	N/A	N/A	N/A	N/A	N/A	N/A	N/A
Wang R	China	202	7	59.43 (47–67)	4 ( 57)	5 (71)	5 (71)	3 (43)	4 (57)	6 (86)	NA	7.5 (5.03–8.02)	0.80 (0.49–0.92)	5.65 (4.29–6.28)	141 (114–213)	5 (71)	N/A	N/A	3 (43)	N/A	N/A	N/A	1 (14)
Tortonese S *	France	44	44	61 (51.5–72.5)	29 (66)	35 (80)	N/A	19 (43)	13 (30)	6 (14)	N/A	N/A	0.6 (0.46–1.04)	3.91 (2.39–6.00)	196.5 (136–249)	0 (0)	3 (7)	4 (9.1)	N/A	41 (93)	3(7)	15 (34)	12 (28)
Alberici F *	Italy	21	21	N/A	N/A	N/A	N/A	N/A	N/A	N/A	N/A	N/A	N/A	N/A	N/A	17 (81) e	17 (81)	1 (5)	4 (19)	21 (100)	0 (0)	0( 0)	N/A
Manganaro M ^	Italy	2893	98	70	58 (59.3)	N/A	N/A	N/A	N/A	N/A	N/A	N/A	N/A	N/A	N/A	N/A	N/A	N/A	N/A	N/A	N/A	N/A	N/A
Scarpioni R	Italy	257	41	73 (52–90)	31 (76)	N/A	N/A	N/A	N/A	N/A	N/A	N/A	N/A	N/A	N/A	N/A	N/A	N/A	N/A	N/A	N/A	N/A	N/A
Esposito P	Italy	260	17	N/A	N/A	N/A	N/A	N/A	N/A	N/A	N/A	N/A	N/A	N/A	N/A	N/A	N/A	N/A	N/A	6 (35)	11 (64)	1 (6)	N/A
Alberici F	Italy	643	94	72 (62–79)	62 (66)	64 (68)	NA	22 (23)	24 (25)	6 (6)	16 (17)	5.08 (3.94–6.48)	0.75 (0.55–1.09)	3.51 (2.69–4.77)	162 (126–230)	60 (64) f	72 (77)	19 (20)	N/A	57 (61)	37 (40)	N/A	57 (61)
Quintaliani G	Italy	30,821	1093	N/A	N/A	N/A	N/A	N/A	N/A	N/A	N/A	N/A	N/A	N/A	N/A	N/A	N/A	N/A	N/A	N/A	N/A	N/A	N/A
La Milia V	Italy	209	55	72.26	N/A	21 (38)	N/A	N/A	N/A	N/A	N/A	N/A	N/A	N/A	N/A	25 (46) e	25 (46)	0	0	25	30	8 (15)
Kikuchi K	Japan	339,841	99	70–90	69 (70)	79 (95) b	N/A	47 (64) b	N/A	N/A	N/A	N/A	N/A	N/A	N/A	31 (31) g	N/A	N/A	15 (15)	99 (100)	0 (0)	N/A	N/A
Cho JH	Korea	1175	11	57 (29–63)	7 (64)	6 (55)	0	2 (18)	0	0	0	N/A	N/A	N/A	N/A	N/A	N/A	N/A	N/A	11 (100)	0 (0)	0( 0)	0
Jung HY *	Korea	14	14	63.5 (40.0–88.0)	6 (43)	N/A	4 (29)	7 (50)	5 (36)	2 (14)	2 (14)	5.8 (4–10)	1.1 (1.0–4.5)	N/A	162.1 (130–400)	14 (100) h	7 (50)	0 (0)	3 (21)	14 (100)	0 (0)	4 (29)	2 (14)
Sánchez-A JE *	Spain	548	548	71 ± 15	359 (66)	416 (76)	N/A	372 (68)	236 (43)	13 (2.3)	N/A	N/A	N/A	N/A	N/A	176 (32) h	396 (72)	12 (2)	80 (14)	444 (81)	104 (19)	33 (6)	N/A
Albalate M	Spain	90	37	67.79 (17–100)	23 (62)	16 (43)	N/A	10 (27)	3 (8)	0 (0)	3 (8)	N/A	0.919 (0.2–1.9)	N/A	N/A	23 (62) i	29 (78)	N/A	N/A	16 (43)	21 (57)	0 (0)	N/A
Goicoechea M	Spain	282	36	71 ± 12	23 (64)	24 (67)	9 (25)	16 (44)	N/A	6(17)	N/A	N/A	0.79 ± 0.47	NA	164 ± 66	2 (5) h	35 (97)	27 (75)	17 (47)	36 (100)	0	1 (2.7)	11 (31)
Sánchez-P P	Spain	478	16	79.5 (73.2–85)	11 (69)	16 (100)	11 (38)	11 (38)	3 (19)	5 (31)	11 (38)	N/A	8.4 (7.3–11.5)	N/A	N/A	0 (0)	12 (75)	2 (13)	4 (33)	16 (100)	0 (0)	2 (13)	N/A
Arslan H	Turkey	602	7	62 (25–79)	3 ( 43)	N/A	N/A	N/A	N/A	N/A	N/A	N/A	N/A	N/A	N/A	N/A	N/A	N/A	N/A	N/A	N/A	N/A	0
Corbett R.W.	UK	1530	300	67 (57–77)	180 (60)	N/A	N/A	N/A	N/A	N/A	N/A	N/A	N/A	N/A	N/A	N/A	N/A	N/A	N/A	N/A	N/A	N/A	N/A
Valeri AM *	USA	59	59	63 (56–78)	33 (56)	29 (49)	13 (22)	23 (39)	21 (36)	9 (15)	4 (7)	6 (4.5–7.8)	0.8 (0.58–1.23)	N/A	N/A	N/A	34 (58)	3 (5)	N/A	59 (100)	0 (0)	8 (14)	N/A
Fisher M *	USA	114	114	64.5	68 (61)	51 (45)	N/A	15 (13)	57 (50)	6 (5)	N/A	5.83	N/A	N/A	N/A	0 (0)	87 (76)	0 (0)	0	114 (100)	0 (0)	15 (13)	14 (12)

Abbreviation: HD: hemodialysis; GI: gastrointestinal symptoms; WBC: White blood cells; HCQ: hydroxychloroquine; CS: Corticosteroids; In: in-patient wards; Out: out-patient clinics; ICU: intensive care units; ARDS: acute respiratory distress syndrome. Age and gender reported statistics of COVID-19 cases. Age was expressed in mean ± SD or median (IQR). The study of Li J et al [13]. reported 2 independent cohorts that were analyzed separately for meta-analysis. * Not included for incidence assessment because the denominator cannot be ascertained from literature or author queries. ^ Not included for mortality assessment because of incomplete data. a. This study reported 154 confirmed COVID-19 cases. Analyses were conducted from 131 cases because 23 patients did not provide consent. The percentages were calculated using 131 cases as denominator. b. This study reported 99 confirmed COVID-19 cases. “Fever” recordings were missing in 16 cases (the percentages were calculated using 83 cases as denominator). "Cough" recordings were missing in 25 cases (the percentages were calculated using 74 cases as denominator). Antiviral therapy was reported as: c. not specified, used 115 patients as denominator; d. used 110 patients as denominator; e. lopinavir/ritonavir or remdesivir; f, lopinavir/ritonavir or darunavir/ritonavir g. famipiravir; h. lopinavir/ritonavir; i. 17 cases using azithromycin and 6 cases using lopinavir/ritonavir.

**Table 2:** Characteristics of studies related to COVID-19 infection in hemodialysis patients.

Authors	Year	Journal	Date (M/D)	Country	City	Study Design	Setting	Duration	Confirmatory Test	Preventive Strategies	Quality Score	Reference
Yau K, et al	2020	Am J Kidney Dis	07/19	Canada	Toronto	Prospective	HD centers	15	RT−PCR	PPE, quarantine, and isolation gowns	13	[14]
Wang H, et al	2020	Kidney Med	04/16	China	Wuhan	Retrospective	Hospital	34	RT-PCR + serology + CT	Timely upgrading of personal protection measures, quarantine and isolation	8	[15]
Su K, et al	2020	Infect Control Hosp Epidemiol	04/24	China	Wuhan	Retrospective	HD centers	26	RT-PCR + serology + CT	Isolation ward, quarantine	9	[16]
Xiong F, et al	2020	J Am Soc Nephrol	05/10	China	Wuhan	Retrospective	Hospital	69	RT−PCR	Medical mask, isolation	14	[17]
Wu J, et al	2020	Clin J Am Soc Nephrol	05/24	China	Wuhan	Retrospective	Hospital	40	RT−PCR	Isolation	13	[18]
Li J, et al (1)	2020	Kidney Disease	05/25	China	Wuhan	Retrospective	Hospital	17	RT−PCR + CT	Isolation in a dedicated area	12	[13]
Li J, et al (2)	2020	Kidney Disease	05/25	China	Wuhan	Retrospective	HD centers	13	RT−PCR + CT	N/A	12	[13]
Wang R, et al	2020	Am J Kidney Dis	05/31	China	Wuhan	Case series	HD centers	N/A	RT−PCR	Surgical or N95 masks	12	[19]
Ma Y, et al	2020	Kidney Int Rep	06/09	China	Wuhan	Retrospective	Hospital	58	RT−PCR+ CT	Patients: N95 mask, quarantine or Isolation. Staff: PPE	12	[20]
Tang H, et al	2020	Am J Kidney Dis	07/03	China	Wuhan	Retrospective	HD centers	121	RT−PCR, serology	N/A	15	[21]
Wang R, et al	2020	Clin Kidney J	07/23	China	Wuhan	Retrospective	Hospital	86	RT−PCR, RT−PCR + CT	Patient: mask. Staff: Waterproof disposable gown, cap, gloves, face shield and N95 face mask	11	[22]
Tortonese S, et al	2020	Kidney Int Rep	07/18	France	Paris	Retrospective	Hospital	61	RT−PCR + CT	Mask	12	[23]
Alberici F, et al	2020	Kidney Int Rep	04/04	Italy	Brescia	Case series	Hospital	N/A	RT−PCR	N/A	11	[24]
Manganaro M, et al	2020	J Nephrol	04/12	Italy	Piedmont/ Aosta Valley	Retrospective	Hospital	35	CXR *	Surgical masks, hand disinfection	8	[25]
Scarpioni R, et al	2020	G Ital Nefrol	04/14	Italy	Piacenza	Retrospective	Hospital	N/A	RT−PCR, CT	Mask, alcohol-based sanitizer, changing clothes and shoes	3	[26]
Esposito P, et al	2020	Hemodial Int	05/05	Italy	Genoa	Retrospective	HD centers	N/A	RT−PCR	Handwashing, use of PPE	9	[27]
Alberici F, et al		Kidney Int	05/08	Italy	Brescia	Retrospective	HD centers	33	RT−PCR	N/A	14	[28]
Quintaliani G, et al	2020	J Nephrol	07/03	Italy	Nationwide	Retrospective	HD centers	59	RT−PCR	N/A	10	[29]
La Milia V, et al	2020	Kidney Int Rep	07/10	Italy	Lombardy	Prospective	Hospital/ HD centers	22	RT−PCR	Upgrade of PPE	11	[30]
Kikuchi K, et al	2020	Ther Apher Dial	06/09	Japan	Nationwide	Prospective	Hospital/ HD centers	89	RT−PCR, CT	Mask, sufficient distance	15	[31]
Cho JH, et al	2020	J Am Soc Nephrol	06/01	Korea	Daegu	Retrospective	HD centers	24	RT−PCR	Mask, hand sanitizer, cohort isolation, notify first	15	[32]
Jung HY, et al	2020	J Clin Med	06/02	Korea	Daegu	Prospective	Hospital	89	RT−PCR	Mask, isolation in negative pressure room	17	[33]
Sánchez-A JE, et al	2020	Nefrologia	04/06	Spain	Nationwide	Prospective	HD centers	24	RT−PCR	N/A	17	[34]
Albalate M, et al	2020	Nefrologia	04/30	Spain	Madrid	Retrospective	Hospital	35	RT−PCR	Mask, alcohol-based sanitizer	10	[35]
Goicoechea M, et al	2020	Kidney Int	05/11	Spain	Madrid	Retrospective	Hospital	29	RT−PCR	N/A	12	[36]
Sánchez-P P, et al	2020	Nefrologia	07/06	Spain	Valencia	Prospective	Hospital/HD centers	45	RT−PCR	PPE, isolation	17	[37]
Arslan H, et al	2020	Exp Clin Transplant	06/11	Turkey	Ankara	Retrospective	HD centers	N/A	RT−PCR + CT	N/A	13	[38]
Corbett RW, et al	2020	J Am Soc Nephrol	06/19	UK	London	Prospective	HD centers	42	RT−PCR	Mask, isolation units	16	[39]
Valeri AM, et al	2020	J Am Soc Nephrol	05/28	USA	New York	Retrospective	Hospital	30	RT−PCR	Staff: Mask, PPE	15	[40]
Fisher M, et al	2020	Kidney360	06/17	USA	New York	Retrospective	Hospital	44	RT−PCR	N/A	14	[41]

Abbreviation: PPE: Personal protective equipment (including masking gloves, face shields, masks, disposable gowns, caps); RT-PCR, Reverse-transcriptase polymerase chain reaction; CT: Chest computed tomography; CXR, Chest X-ray; Ref, reference. * COVID-19 infection was confirmed if signs of interstitial pneumonia presented on chest radiography, nucleic acid testing was performed only in person having contact history with suspected or confirmed cases of COVID-19. Duration: denoted observation period, expressed in days. Quality score indicates the number of positive answers in the case-series appraisal sheets. The study of Li J et al. reported two independent cohorts, which were analyzed separately for meta-analysis.

## Data Availability

Not applicable.

## Supplementary Materials

The following are available online at https://www.mdpi.com/2227-9032/9/1/47/s1, Table S1: Search strategy, Table S2: PRISMA checklist.

## References

[1] WHO (2020). Coronavirus Disease (COVID-19) Dashboard. https://covid19.who.int.

[2] McMichael T.M., Currie D.W., Clark S., Pogosjans S., Kay M., Schwartz N.G., Lewis J., Baer A., Kawakami V., Lukoff M.D. (2020). Epidemiology of Covid-19 in a Long-Term Care Facility in King County, Washington. N. Engl. J. Med..

[3] Zhou F., Yu T., Du R., Fan G., Liu Y., Liu Z., Xiang J., Wang Y., Song B., Gu X. (2020). Clinical course and risk factors for mortality of adult inpatients with COVID-19 in Wuhan, China: A retrospective cohort study. Lancet.

[4] Rao K.S., Suryaprakash V., Senthilkumar R., Preethy S., Katoh S., Ikewaki N., Abraham S.J.K. (2020). Role of immune dysregulation in increased mortality among a specific subset of COVID-19 patients and immune-enhancement strategies for combatting through nutritional supplements. Front. Immunol..

[5] GBD Chronic Kidney Disease Collaboration (2020). Global, regional, and national burden of chronic kidney disease, 1990-2017: A systematic analysis for the Global Burden of Disease Study 2017. Lancet.

[6] Carney E.F. (2020). The impact of chronic kidney disease on global health. Nat. Rev. Nephrol..

[7] USRDS (2019). US renal data system 2018 annual data report. Am. J. Kidney Dis..

[8] Betjes M.G. (2013). Immune cell dysfunction and inflammation in end-stage renal disease. Nat. Rev. Nephrol..

[9] Henry B.M., Lippi G. (2020). Chronic kidney disease is associated with severe coronavirus disease 2019 (COVID-19) infection. Int. Urol. Nephrol..

[10] Moher D., Liberati A., Tetzlaff J., Altman D.G. (2009). Preferred reporting items for systematic reviews and meta-analyses: The PRISMA statement. PloS Med..

[11] Alqahtani J.S., Oyelade T., Aldhahir A.M., Alghamdi S.M., Almehmadi M., Alqahtani A.S., Quaderi S., Mandal S., Hurst J.R. (2020). Prevalence, severity and mortality associated with copd and smoking in patients with COVID-19: A rapid systematic review and meta-analysis. PLoS ONE.

[12] Liu C., He Y., Liu L., Li F., Shi Y. (2020). Children with COVID-19 behaving milder may challenge the public policies: A systematic review and meta-analysis. BMC Pediatr..

[13] Li J., Yang Y., Gong M., Shi J., Zhou X., Xing X., Pan H., Guo S., Chang X., Cheng A. (2020). Aggressive Quarantine Measures Reduce the High Morbidity of COVID-19 in Patients on Maintenance Hemodialysis and Medical Staff of Hemodialysis Facilities in Wuhan, China. Kidney Dis..

[14] Yau K., Muller M.P., Lin M., Siddiqui N., Neskovic S., Shokar G., Fattouh R., Matukas L.M., Beaubien-Souligny W., Thomas A. (2020). COVID-19 Outbreak in an Urban Hemodialysis Unit. Am. J. Kidney Dis..

[15] Wang H. (2020). Maintenance hemodialysis and COVID-19: Saving lives with caution, care, and courage. Kidney Med..

[16] Su K., Ma Y., Wang Y., Song Y., Lv X., Wei Z., Shi M., Ding G., Shen B., Wang H. (2020). How we mitigated and contained the COVID-19 outbreak in a hemodialysis center: Lessons and experience. Infect. Control Hosp. Epidemiol..

[17] Xiong F., Tang H., Liu L., Tu C., Tian J.B., Lei C.T., Liu J., Dong J.W., Chen W.L., Wang X.H. (2020). Clinical Characteristics of and Medical Interventions for COVID-19 in Hemodialysis Patients in Wuhan, China. J. Am. Soc. Nephrol..

[18] Wu J., Li J., Zhu G., Zhang Y., Bi Z., Yu Y., Huang B., Fu S., Tan Y., Sun J. (2020). Clinical Features of Maintenance Hemodialysis Patients with 2019 Novel Coronavirus-Infected Pneumonia in Wuhan, China. Clin. J. Am. Soc. Nephrol..

[19] Wang R., Liao C., He H., Hu C., Wei Z., Hong Z., Zhang C., Liao M., Shui H. (2020). COVID-19 in Hemodialysis Patients: A Report of 5 Cases. Am. J. Kidney Dis..

[20] Ma Y., Diao B., Lv X., Zhu J., Chen C., Liu L., Zhang S., Shen B., Wang H. (2020). Epidemiological, Clinical, and Immunological Features of a Cluster of COVID-19-Contracted Hemodialysis Patients. Kidney Int. Rep..

[21] Tang H., Tian J.B., Dong J.W., Tang X.T., Yan Z.Y., Zhao Y.Y., Xiong F., Sun X., Song C.X., Xiang C.G. (2020). Serologic Detection of SARS-CoV-2 Infections in Hemodialysis Centers: A Multicenter Retrospective Study in Wuhan, China. Am. J. Kidney Dis..

[22] Wang R., He H., Liao C., Hu H., Hu C., Zhang J., Gao P., Wu X., Cheng Z., Liao M. (2020). Clinical outcomes of hemodialysis patients infected with severe acute respiratory syndrome coronavirus 2 and impact of proactive chest computed tomography scans. Clin. Kidney J..

[23] Tortonese S., Scriabine I., Anjou L., Loens C., Michon A., Benabdelhak M., Ouali S., Morin G., Laifi M., Dobosziewicz H. (2020). COVID-19 in Patients on Maintenance Dialysis in the Paris Region. Kidney Int. Rep..

[24] Alberici F., Delbarba E., Manenti C., Econimo L., Valerio F., Pola A., Maffei C., Possenti S., Piva S., Latronico N. (2020). Management of patients on dialysis and with kidney transplantation during the SARS-CoV-2 (COVID-19) pandemic in Brescia, Italy. Kidney Int. Rep..

[25] Manganaro M., Baldovino S., Working Group of the Piedmont, Aosta Valley Section of the SI.N (2020). First considerations on the SARS-CoV-2 epidemic in the Dialysis Units of Piedmont and Aosta Valley, Northern Italy. J. Nephrol..

[26] Scarpioni R., Manini A., Valsania T., De Amicis S., Albertazzi V., Melfa L., Ricardi M., Rocca C. (2020). Covid-19 and its impact on nephropathic patients: The experience at Ospedale "Guglielmo da Saliceto" in Piacenza. G. Ital. Nefrol..

[27] Esposito P., Russo R., Conti N., Falqui V., Massarino F., Moriero E., Peloso G., Traverso G.B., Garibotto G., Viazzi F. (2020). Management of COVID-19 in hemodialysis patients: The Genoa experience. Hemodial. Int..

[28] Alberici F., Delbarba E., Manenti C., Econimo L., Valerio F., Pola A., Maffei C., Possenti S., Lucca B., Cortinovis R. (2020). A report from the Brescia Renal COVID Task Force on the clinical characteristics and short-term outcome of hemodialysis patients with SARS-CoV-2 infection. Kidney Int..

[29] Quintaliani G., Reboldi G., Di Napoli A., Nordio M., Limido A., Aucella F., Messa P., Brunori G., Italian Society of Nephrology, C.-R.G. (2020). Exposure to novel coronavirus in patients on renal replacement therapy during the exponential phase of COVID-19 pandemic: Survey of the Italian Society of Nephrology. J. Nephrol..

[30] La Milia V., Bacchini G., Bigi M.C., Casartelli D., Cavalli A., Corti M., Crepaldi M., Limardo M., Longhi S., Manzoni C. (2020). COVID-19 Outbreak in a Large Hemodialysis Center in Lombardy, Italy. Kidney Int. Rep..

[31] Kikuchi K., Nangaku M., Ryuzaki M., Yamakawa T., Hanafusa N., Sakai K., Kanno Y., Ando R., Shinoda T., Nakamoto H. (2020). COVID-19 of dialysis patients in Japan: Current status and guidance on preventive measures. Ther. Apher. Dial..

[32] Cho J.H., Kang S.H., Park H.C., Kim D.K., Lee S.H., Do J.Y., Park J.W., Kim S.N., Kim M.S., Jin K. (2020). Hemodialysis with Cohort Isolation to Prevent Secondary Transmission during a COVID-19 Outbreak in Korea. J. Am. Soc. Nephrol..

[33] Jung H.Y., Lim J.H., Kang S.H., Kim S.G., Lee Y.H., Lee J., Chang H.H., Kim S.W., Choi J.Y., Cho J.H. (2020). Outcomes of COVID-19 among Patients on In-Center Hemodialysis: An Experience from the Epicenter in South Korea. J. Clin. Med..

[34] Sánchez-Álvarez J.E., Pérez Fontán M., Jiménez Martín C., Blasco Pelícano M., Cabezas Reina C.J., Sevillano Prieto Á.M., Melilli E., Crespo Barrios M., Macía Heras M., Del Pino Y.P.M.D. (2020). SARS-CoV-2 infection in patients on renal replacement therapy. Report of the COVID-19 Registry of the Spanish Society of Nephrology (SEN). Nefrologia.

[35] Albalate M., Arribas P., Torres E., Cintra M., Alcazar R., Puerta M., Ortega M., Procaccini F., Martin J., Jimenez E. (2020). High prevalence of asymptomatic COVID-19 in haemodialysis: Learning day by day in the first month of the COVID-19 pandemic. Nefrologia.

[36] Goicoechea M., Sanchez Camara L.A., Macias N., Munoz de Morales A., Rojas A.G., Bascunana A., Arroyo D., Vega A., Abad S., Verde E. (2020). COVID-19: Clinical course and outcomes of 36 hemodialysis patients in Spain. Kidney Int..

[37] Sánchez-Pérez P., González-Calero P., Poma-Saavedra F.H., Orero-Calvé E., Devesa-Such R., Soldevila-Orient A., Henningsmeyer-Utrera B., Lacueva-Moya J. (2020). Results of a healthcare organization model for COVID-19 on hemodialysis in a tertiary hospital and its subsidized centers. Nefrologia.

[38] Arslan H., Musabak U., Ayvazoglu Soy E.H., Kurt Azap O., Sayin B., Akcay S., Haberal K.M., Akdur A., Yildirim S., Haberal M. (2020). Incidence and Immunologic Analysis of Coronavirus Disease (COVID-19) in Hemodialysis Patients: A Single-Center Experience. Exp. Clin. Transplant..

[39] Corbett R.W., Blakey S., Nitsch D., Loucaidou M., McLean A., Duncan N., Ashby D.R., West London R., Transplant C. (2020). Epidemiology of COVID-19 in an Urban Dialysis Center. J. Am. Soc. Nephrol..

[40] Valeri A.M., Robbins-Juarez S.Y., Stevens J.S., Ahn W., Rao M.K., Radhakrishnan J., Gharavi A.G., Mohan S., Husain S.A. (2020). Presentation and Outcomes of Patients with ESKD and COVID-19. J. Am. Soc. Nephrol..

[41] Fisher M., Yunes M., Mokrzycki M.H., Golestaneh L., Alahiri E., Coco M. (2020). Chronic hemodialysis patients hospitalized with COVID-19: Short-term outcomes in the bronx, New York. Kidney360.

[42] To K.K.-W., Tsang O.T.-Y., Leung W.-S., Tam A.R., Wu T.-C., Lung D.C., Yip C.C.-Y., Cai J.-P., Chan J.M.-C., Chik T.S.-H. (2020). Temporal profiles of viral load in posterior oropharyngeal saliva samples and serum antibody responses during infection by SARS-CoV-2: An observational cohort study. Lancet Infect. Dis..

[43] Anand S., Montez-Rath M., Han J., Bozeman J., Kerschmann R., Beyer P., Parsonnet J., Chertow G.M. (2020). Prevalence of SARS-CoV-2 antibodies in a large nationwide sample of patients on dialysis in the USA: A cross-sectional study. Lancet.

[44] Mehra M.R., Desai S.S., Kuy S., Henry T.D., Patel A.N. (2020). Cardiovascular Disease, Drug Therapy, and Mortality in Covid-19. N. Engl. J. Med..

[45] Cummings M.J., Baldwin M.R., Abrams D., Jacobson S.D., Meyer B.J., Balough E.M., Aaron J.G., Claassen J., Rabbani L.E., Hastie J. (2020). Epidemiology, clinical course, and outcomes of critically ill adults with COVID-19 in New York City: A prospective cohort study. Lancet.

[46] Mehta P., McAuley D.F., Brown M., Sanchez E., Tattersall R.S., Manson J.J. (2020). HLH Across Speciality Collaboration, UK. COVID-19: Consider cytokine storm syndromes and immunosuppression. Lancet.

[47] Wiersinga W.J., Rhodes A., Cheng A.C., Peacock S.J., Prescott H.C. (2020). Pathophysiology, Transmission, Diagnosis, and Treatment of Coronavirus Disease 2019 (COVID-19): A Review. JAMA.

[48] Henry B.M., de Oliveira M.H.S., Benoit S., Plebani M., Lippi G. (2020). Hematologic, biochemical and immune biomarker abnormalities associated with severe illness and mortality in coronavirus disease 2019 (COVID-19): A meta-analysis. Clin. Chem. Lab. Med..

[49] Tzotzos S.J., Fischer B., Fischer H., Zeitlinger M. (2020). Incidence of ARDS and outcomes in hospitalized patients with COVID-19: A global literature survey. Crit. Care.

[50] Roberts M.A., Velkoska E., Ierino F.L., Burrell L.M. (2013). Angiotensin-converting enzyme 2 activity in patients with chronic kidney disease. Nephrol. Dial. Transplant..

[51] Bourgonje A.R., Abdulle A.E., Timens W., Hillebrands J.L., Navis G.J., Gordijn S.J., Bolling M.C., Dijkstra G., Voors A.A., Osterhaus A.D. (2020). Angiotensin-converting enzyme 2 (ACE2), SARS-CoV-2 and the pathophysiology of coronavirus disease 2019 (COVID-19). J. Pathol..

[52] Roche J.A., Roche R. (2020). A hypothesized role for dysregulated bradykinin signaling in COVID-19 respiratory complications. FASEB J..

