# Supplementary material for: Incidence and Clinical Impacts of COVID-19 Infection in Patients with Hemodialysis: Systematic Review and Meta-Analysis of 396,062 Hemodialysis Patients
Source: Healthcare (Basel). 2021 Jan 5;9(1):47. doi: 10.3390/healthcare9010047 (PMC7824817; doi:10.3390/healthcare9010047)
Supplement: Supplementary file 1 [file healthcare-09-00047-s001.zip › Table S1_search strategy.docx]

| **Table S1. Search strategy** |
| --- |
| **PUBMED** |
| COVID-19 AND (hemodialysis) AND (incidence or prevalence or mortality or prognosis) |
| **EMBASE** |
| 'coronavirus disease 2019'/exp AND ('hemodialysis'/exp OR 'blood dialysis' OR 'chronic haemodialysis' OR 'chronic hemodialysis' OR 'chronic intermittent haemodialysis' OR 'chronic intermittent hemodialysis' OR 'dialysis center' OR 'dialysis, blood' OR 'extracorporeal blood cleansing' OR 'extracorporeal dialysis' OR 'haemodialysis' OR 'haemodialysis center' OR 'haemodialysis centre' OR 'haemodialysis department' OR 'haemodialysis unit' OR 'haemodialysis units, hospital' OR 'hemodialyse' OR 'hemodialysis' OR 'hemodialysis center' OR 'hemodialysis department' OR 'hemodialysis unit' OR 'hemodialysis units, hospital' OR 'hemorenodialysis' OR 'hemotrialysate' OR 'intermittent chronic haemodialysis' OR 'intermittent chronic hemodialysis' OR 'intermittent haemodialysis' OR 'intermittent hemodialysis' OR 'renal dialysis') AND ('incidence'/exp OR 'prevalence'/exp OR 'mortality'/exp OR 'prognosis'/exp) |
